# Automated design of paralogue ratio test assays for the accurate and rapid typing of copy number variation

**DOI:** 10.1093/bioinformatics/btt330

**Published:** 2013-06-06

**Authors:** Colin D. Veal, Hang Xu, Katherine Reekie, Robert Free, Robert J. Hardwick, David McVey, Anthony J. Brookes, Edward J. Hollox, Christopher J. Talbot

**Affiliations:** ^1^Department of Genetics, University of Leicester, Leicester, LE1 7RH and ^2^Department of Cardiovascular Sciences, University of Leicester, LE3 9QP, UK

## Abstract

**Motivation:** Genomic copy number variation (CNV) can influence susceptibility to common diseases. High-throughput measurement of gene copy number on large numbers of samples is a challenging, yet critical, stage in confirming observations from sequencing or array Comparative Genome Hybridization (CGH). The paralogue ratio test (PRT) is a simple, cost-effective method of accurately determining copy number by quantifying the amplification ratio between a target and reference amplicon. PRT has been successfully applied to several studies analyzing common CNV. However, its use has not been widespread because of difficulties in assay design.

**Results:** We present PRTPrimer (www.prtprimer.org) software for automated PRT assay design. In addition to stand-alone software, the web site includes a database of pre-designed assays for the human genome at an average spacing of 6 kb and a web interface for custom assay design. Other reference genomes can also be analyzed through local installation of the software. The usefulness of PRTPrimer was tested within known CNV, and showed reproducible quantification. This software and database provide assays that can rapidly genotype CNV, cost-effectively, on a large number of samples and will enable the widespread adoption of PRT.

**Availability:** PRTPrimer is available in two forms: a Perl script (version 5.14 and higher) that can be run from the command line on Linux systems and as a service on the PRTPrimer web site (www.prtprimer.org).

**Contact:** cjt14@le.ac.uk

**Supplementary Information:** Supplementary data are available at *Bioinformatics* online.

## 1 INTRODUCTION

Copy number variation (CNV) is a pervasive and extensive source of variation between individual genomes in humans and many other species. A genome-wide picture of CNV has been provided in humans by large consortia, typically using array-comparative genomic hybridization (Conrad *et al.*, 2010; Itsara *et al.*, 2009; Pang *et al.*, 2010). For example, a key analysis used an oligonucleotide tiling array to assay common CNV of >1 kb and found that two diploid human genomes typically differed in >1000 CNVs, covering a length of 24 Mb (0.8% of the genome). For the 41 individuals studied from two populations taken together, 3.7% of the genome was shown to be copy number variable (Conrad *et al.*, 2010). In another study, Itsara *et al.* studied large, rare CNV and showed that 65–80% of individuals have a CNV of >100 kb (Itsara *et al.*, 2009). Pang *et al*. constructed a comprehensive map of CNV in a single genome and estimated structural variation to cover 48.8 Mb, a region including 4867 genes (Pang *et al.*, 2010).

There is considerable evidence for CNV affecting phenotype, much of which comes from disease studies in humans. Association analysis of common CNV with phenotypes is limited, in part, by the inherent noise in typing complex CNV with aCGH technology or quantitative real-time PCR (qPCR). Nevertheless, the Wellcome Trust Case Control Consortium (WTCCC) analyzed the genome-wide association between common CNV (56% had minor allele frequency >5%) and reported five robust associations with complex diseases (Even though they included only 42–50% of CNV with a minor allele frequency >5%) (The Wellcome Trust Case Control Consortium, 2010). Other well-founded associations include the effect of high β-defensin copy number on psoriasis (Hollox *et al.*, 2008; Stuart *et al.*, 2012), the effect of *CYP2D6* copy number on drug metabolism (Zhou *et al.*, 2009) and the role of α-globin copy number in α-thalassemia and protection against severe malaria (May *et al.*, 2007). Rare CNVs have also been shown to have major effects in human disease, particularly in neurological disorders such as schizophrenia and autism (Lee *et al.*, 2011; Morrow, 2010), although methodological issues have complicated interpretation. In *Drosophila melanogaster*, the *Cyp6g1* gene copy number is associated with resistance to the insecticide dichlorodiphenyltrichloroethane (Schmidt *et al.*, 2010), and amplification of the *pfmdr1* gene confers mefloquine resistance in *Plasmodium falciparum* (Cowman *et al.*, 1994). In dogs, the diploid copy number of *AMY2B* ranges between 4 and 30, and may be involved in the adaption to a starch-rich diet in early domestication (Axelsson *et al.*, 2013). These both provide compelling evidence for a functional effect of CNV in other species.

To maximize the power of association studies of CNV and phenotype, it is necessary to characterize CNV in terms of the actual integer number of copies per diploid genome (1, 2, 3, 4 etc.) in a cost-effective manner on large numbers of DNA samples. Genome-wide genotyping of CNV is possible by aCGH, sequence read depth analysis of next-generation sequencing and analysis of hybridization signal intensity from SNP genotyping arrays (Sudmant *et al.*, 2010). These methods can detect CNV, but are limited in their ability to accurately call integer copy number of complex multiallelic CNVs. A comprehensive comparison of array platforms and CNV detection software showed <50% concordance between approaches and reproducibility of replicate experiments at <70% (Pinto *et al.*, 2011). For example, current sequence read depth analysis methods have high false discovery rates (<10–89%), relatively low detection rates (30–60%) and are crucially dependent on the quality of read alignment to the reference genome (Abyzov *et al.*, 2011; Mills *et al.*, 2011; Teo *et al.*, 2012). In addition, micrograms of DNA are often required by these methods, with whole genome amplification not being viable, as current methods are known to introduce inconsistent copy number bias (Pugh *et al.*, 2008).

There are three commonly used locus-specific methods that attempt to meet the challenge of accurate and efficient genotyping of CNV in large numbers of samples: multiplex ligation-dependent amplification, qPCR and the paralogue ratio test (PRT). The multiplex ligation-dependent amplification method is used routinely in clinical genetics for screening disease genes for exons, and has been shown to accurately infer integer copy number even on challenging multicopy loci (Eijk-Van Os and Schouten, 2011). However, it is propriety, expensive and commercially available only for certain loci, and often 100–200 ng of DNA is required. qPCR is frequently used as a high-throughput method for CNV typing, particularly using fluorescent-quencher systems such as TaqMan. However, the method is susceptible to variation in DNA quality and often gives apparently precise, but inaccurate, results at multicopy loci. This has been a bane of human CNV studies, leading to controversy in the field (Cantsilieris and White, 2013; Perne *et al.*, 2009).

PRT is a form of quantitative PCR that differs from the conventional method in one important aspect: by careful design of the primers targeting paralogous sequences, one primer pair is used to amplify a putative CNV target locus relative to a single copy reference locus (Armour *et al.*, 2007). This makes the kinetics of target and reference amplification similar, and results in increased accuracy of integer copy number calling. The target and reference amplicons can be separated by size using electrophoresis or by sequence using pyrosequencing, restriction enzyme digest or real-time PCR with sequence-specific fluorescent reporter probes. PRT assays have been successfully applied in a number of human CNV studies (Aldhous *et al.*, 2010; Carpenter *et al.*, 2011; Fode *et al.*, 2011a, b; Hollox *et al.*, 2009; Morris *et al.*, 2010; Saldanha *et al.*, 2011). Currently, PRT assay design involves a laborious process of selecting either a self-chain segment (a short sequence that matches to more than one place in the genome, but is not a known repeat element) or a low copy number repeat within the target sequence. The selected sequence is then mapped to the reference genome using BLAT (Kent, 2002), and the sequence alignment of the target and paralogue(s) examined to identify two identically matched sequences long enough to allow primer design and flanking a stretch of DNA either of different length or sequence. Last, the putative primers are assessed using *in silico* PCR, to check that they produce only two amplicons of the predicted size, failing which the process will need to be repeated.

This current design approach requires several hours for each assay and is dependent on self-chain or a low copy number repeat sequence in the target interval, which limits the number of assays that can be designed. In addition, there is some probability that an assay will not successfully transfer into the laboratory. These problems have prevented the widespread adoption of PRT, despite its benefits over other technologies, but could be overcome by an automated approach to assay design.

With this in mind, we have developed the software PRTPrimer and the web resource www.prtprimer.org. PRTPrimer is aimed at all users who would benefit from designing PRT assays for the human genome. The software can be installed locally or run through the web resource, is optimized for multicore systems and can be adapted to use genomes from other species.

## 2 SOFTWARE

### 2.1 Features

**Fig. 1.**
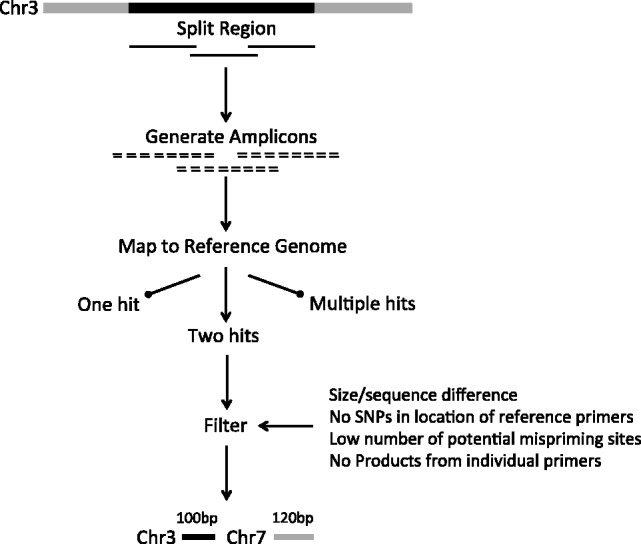
Overview of PRTPrimer. Target region for which PRTs are required on chromosome 3 is shown in black. The software first splits the region into overlapping segments to ensure an even distribution of PRTs. A large number of amplicons are designed for each of these segments irrespective of what type of sequence they are in, i.e. segmental duplication, SINE and so forth. Each amplicon is then aligned to the human genome allowing for mismatches with the primers. Only amplicons that have exact priming matches twice in the genome are selected for filtering. The final stage filters the results to only those amplicons that meet adjustable criteria, such as size difference between target and reference, no SNPs at primer positions and CNVs spanning the reference amplicon. In this example, the target amplicon is 100 bp and the reference amplicon on a different chromosome is 120 bp

We have devised an automated approach to PRT design that uses brute-force computation based on the following steps (Fig. 1).

1. Design a large number of primer pairs in the target interval.
2. Determine the location of potential amplification sites of these primer pairs in the reference human genome.
3. Isolate those that are perfect priming matches for only two amplicons in the reference genome.
4. Apply filtering to identify optimal PRT assays for the target region.

PRTPrimers are available in two forms: a Perl script (version 5.14 and higher) that can be run from the command line on Linux systems and as a service on the PRTPrimer web site (www.prtprimer.org). The software takes genomic coordinates (GRCh37) or sequence in FASTA format (command line only), and outputs a file of potential PRT assays.

### 2.2 Input options

PRT assay accuracy is dependent on the equally efficient amplification of the target and reference amplicons. Later in the article, we describe parameters that allow these amplicons to be designed in most genomic regions. A summary of all parameters is available in Supplementary Table S1.

### 2.3 Masking

By default PRTPrimer uses a set of sequence masking options:

- SNP masking (dbSNP build 135). This reduces potential amplification differences between individuals due to allelic differences affecting primer annealing.
- Alu masking [RepeatMasker (www.repeatmasker.org)]. Primers for PRT assays that are within Alu elements are more difficult to optimize for copy number calling because of the high copy number of Alu elements.
- Simple tandem repeat masking [Tandem Repeat Finder (Benson, 1999)]. Primers within simple tandem repeats (STRs) have the potential to misalign within the repeat or anneal to similar STRs elsewhere in the genome.

Each of these masking options can be toggled for regions where PRTs are not being designed under default masking.

### 2.4 Primer design

For any particular genomic region, primer pairs are more likely to amplify a single amplicon from a unique region or amplify many amplicons from a multicopy region, rather than amplify two products from separate regions. It is the latter condition that is required for a PRT, and to increase the chance that suitable PRTs will be identified, the algorithm generates many primer pairs (e.g. 2000 per kb) within the target interval. The number of amplicons designed for the interval is controlled by the parameter ppn, which determines the average number of primer pairs to be designed per nucleotide (default 2). The algorithm also limits the amplicon size range (default 100–300 bp) and sets optimal/min/max primer lengths. We used the popular and reliable Primer3 program (Rozen and Skaletsky, 2000) to design the primer pairs. Primer3 is provided as a complete package as OpenSource from http://primer3.sourceforge.net/. The initial Primer3 amplicon design is as close to the optimum parameters as possible, for each subsequent design the optimum parameters are relaxed in decrements until the set number of primer pairs has been reached. This concentration of primers within sequences close to the optimal parameters can cause a poor distribution of amplicons across the target interval. To ensure a more even distribution, PRTPrimer splits the input sequence into overlapping segments in which Primer3 is separately run (default is 2 kb, offset by 300 bp). Additional Primer3 parameters can be edited directly in the Perl script.

### 2.5 Alignment to reference genome

The algorithm generates a large number of primer pairs, dependent on the size of the target region and the value of ppn. To ensure an efficient alignment algorithm, we selected isPCR, which uses the BLAT algorithm to create an index of 11mers from the genome to increase alignment speed (Kent, 2002). isPCR can be obtained free from http://users.soe.ucsc.edu/∼kent/ for academic, non-profit and personal use. This aligns each primer to the reference genome and determines whether a product under a specified length could be amplified. Using default parameters (tileSize = 11, stepSize = 5, minPerfect = 15), isPCR identifies all exact matches in the reference genome and a proportion of products derived from mispriming sites, i.e. nucleotide mismatches between primer and reference genome. An optional ‘sensitive’ setting (tileSize = 10, stepSize = 1, minPerfect = 5) detects a greater proportion of potential mispriming sites; however, this can take a substantially longer time, up to 10-fold, to process depending on the size and sequence composition of the primers.

### 2.6 Filtering

**Fig. 2.**
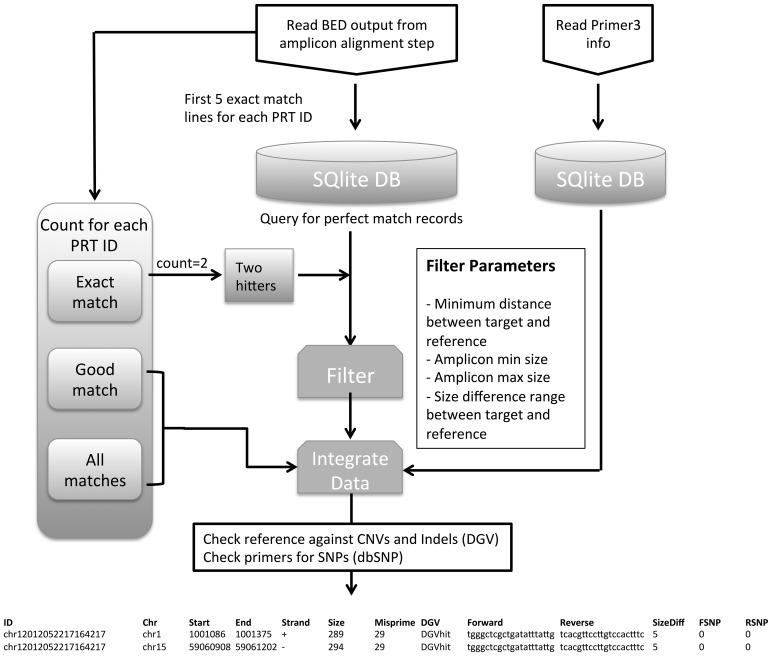
The output of the alignment procedure is stored in a text file [BED format (genome.ucsc.edu/FAQ/FAQformat.html)] with the PRT ID, genomic location and alignment score. Because of the potential size of these files, SQLite databases are used to aid efficient access to these files without prohibitive memory requirements. The original output from Primer3 is stored in a single database and the BED files are processed to store a maximum of the first five exact genomic matches for each amplicon. In addition, each amplicon is counted for the total number of exact matches, and matches with an alignment score >900 (indicating up to four mismatches). The characteristics of amplicons that match just twice in the genome and their corresponding Primer3 data can be rapidly extracted from these databases. The results can then be filtered for various output settings and checked for existing CNV, indels and SNPs. The final output can be seen at the bottom for a single PRT: ID = unique ID for PRT; Chr, Start, End = genomic location; Size = amplicon length in bp; Misprime = potential number of genomic locations that may be amplified with small number of mismatches in primers; DGV = detects whether the amplicon coincides with any reported CNV or indels; Forward, Reverse = amplicon primers; SizeDiff = length difference between target and reference amplicons; FSNP, RSNP = number of SNPs within primers

The data generated by the alignment stage can be substantial, as the data include all primer pair perfect matches, which could be from 1 to many 1000s, as well as the amplicon locations from potential mispriming. The software filters these data to include only those primers that produce two amplicons with exact primer matches. This is illustrated in Figure 2 and described further in ‘implementation and performance’. PRT products can be separated and quantified by different technologies, and as such a number of filtering options are available to the user to select those most appropriate for their technology of choice.

1. Minimum/maximum size difference between the target and reference amplicon. The simplest way to distinguish the PRT amplicons is through a size difference. A size difference of zero can be selected if the user wishes to search for RFLPs or qPCR probes within the products; a size difference of >2 is recommended for detection by capillary electrophoresis; and a size difference of ∼10% of the maximum amplicon size is recommend for detection by agarose gel electrophoresis.
2. Minimum/maximum product size of reference amplicon (also applies to target amplicon, but only if these values are within the limits set for Primer3). Very small or very large amplicons may be undesirable with some technologies, but can be useful if there is limited PRT choice.
3. Minimum distance between target and reference (default 500 kb). This prevents the references being within the boundaries of the target CNV.

### 2.7 Output

Each final potential PRT is displayed on two lines, one for the target and one for the reference (example at the bottom of Fig. 2). In each case, the amplicon genomic coordinates and size are followed by additional information to aid PRT selection:

1. Number of detected potential misprimed amplicons with an isPCR score >900 (representing ∼4 mismatches between primers and hybridization site). A low number for this count indicates that there would be a high probability of only two amplicons being produced, with only a small probability of incorrect products being amplified.
2. Indication whether amplicons lay within an annotated CNV or indel [annotation according to the database of genomic variants (DGV)]. This might be expected for target, but should be avoided for reference, with the caveat that exact boundaries and validity of many of the CNVs in DGV are open to question.
3. Indication of SNPs in primers at target and reference (dbSNP build 135). This will only apply for reference if the target was designed with SNP masking. This checks against all SNPs in dbSNP, including rare and unvalidated variants.

### 2.8 Special modifications

#### 2.8.1 Multitarget CNV

For some copy number variants, the target sequence is present in the reference genome more than once per haploid genome. The standard method of searching for two amplicons in the reference genome does not take this into account. A modification has been applied to the program that allows up to four specified targets in the reference genome. The software will check that the PRT amplifies within all these targets plus a reference with a different size to the amplicons in the targets.

#### 2.8.2 Other species

The Perl script can be easily modified for other organisms, and has been tested with rhesus macaque and mouse genomes.

### 2.9 Web site

The PRTPrimer.org web site has been designed to allow users to take advantage of PRTPrimer without the need for local installation. There is also searchable database of a genome-wide set of pre-designed PRTs.

### 2.10 Genome-wide pre-design database

We have performed a genome-wide search for PRTs (hg19, masked for SNPs, STRs and Alu elements) using 50 Mb segments of the genome, each further split into 2 kb fragments overlapping by 300 bp, to give an average of one primer pair per nucleotide. Each PRT was further assessed using the sensitive settings of isPCR to find all potential mispriming sites and checked for amplicon generation from a single primer. This database can be searched by location, size difference and maximum potential misprimed amplicons. The output, example in Supplementary Figure S1, can be sorted by each column and can be output in a variety of formats. There are also hyperlinks to locate the position of target and reference for a PRT on the UCSC genome browser. The returned PRTs can be displayed as a custom track on the UCSC genome browser (Supplementary Fig. S2).

### 2.11 Live design

If there are no suitable pre-designed PRTs in a target region, the user can perform a custom PRT search on the web site. Jobs are queued on a first-come first-served basis and results are emailed to the user along with a link to a custom track on the UCSC genome browser.

## 3 RESULTS

### 3.1 Implementation and performance

PRTPrimer is written in Perl (www.perl.org) and should run on any linux system with at least Perl 5.14.1, SQLite and 12 GB of RAM. Package and installation instructions are available at www.PRTPrimer.org. The online version has been tested on all modern browsers, including Chrome, Safari, Internet Explorer and Firefox.

A major consideration for PRTPrimer was speed, as the searching of large numbers of primers against the genome is computationally intensive. We optimized the script to take advantage of modern multicore processors by running the isPCR in eight threads in parallel; however, this resulted in increasing the memory requirement to 12 GB of RAM. The script can be adapted to run on systems with higher or lower numbers of processing cores with corresponding increases or decreases in memory usage. SQLite was implemented to handle the large arrays of primer data that could not be held in memory. As PRTPrimer is written in Perl, it is relatively simple for any user to edit the program for different RAM/CPU systems or to add additional species.

### 3.2 Parameter refinement

**Fig. 3.**
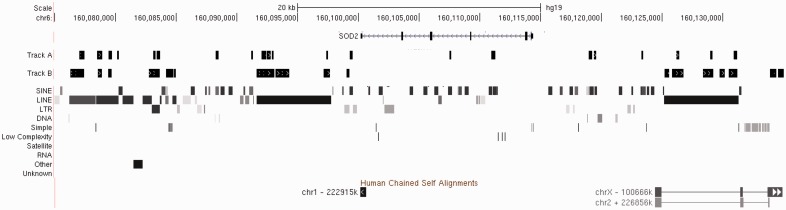
A diagram from the UCSC Genome Browser of PRT assays designed for the *SOD2* gene region under two different runs of PRTPrimer using different parameters aligned against RepeatMasker and self-chain output for the interval. For Track A, primers were allowed to be designed in SINES and other genome regions. For Track B, SINES were excluded from the designed process. Track B resulted in PRT assays that are more likely to succeed in the laboratory, whereas Track A has a higher density of coverage

To optimize parameter defaults we have experimented with different design parameters: potential to misprime due to tolerance of mismatched nucleotides in the primers, masking of high copy number sequences [e.g. Short INterspersed Elements (SINEs)] and primer density. Experiments were conducted on the human genome (GRCh37/hg19), using two targets on chromosomal regions 6p25 and 21q21. Figure 3 shows the results from PRTPrimer under two different settings for a region around the *SOD2* gene (Chr6) that had previously been unsuccessful for manual design. Track A assays are mostly within SINEs and LINEs, whereas Track B assays are in LINEs, LTRs and human self-chain segments. Track A shows results for 200 assays in 21 clusters, and Track B shows 1823 assays in 15 clusters. To examine the reliability of PRT assays we amplified eight PRT assays, group A, that had a high potential for mispriming (>3 products generated from <4 nucleotide mismatches in the primers) and nine PRT assays (seven from Track A and two from Track B), group B, that had a low potential for mispriming (<3 products generated from <4 nucleotide mismatches in the primers). Of the group B assays, four showed two clean PCR products and consistent copy number calls, whereas all group A assays failed to produce clean PCR products. This suggested that assays designed in SINEs have a low success rate in the laboratory because of the high number of mispriming matches in the genome. Approximately 25% of Track A assays and 39% of Track B assays had a low potential for mispriming, indicating that the likely conversion rate for PRTs generated on masked sequence will be higher than unmasked sequence. An example PRT generated by PRTPrimer can be seen in Supplementary Figure S3. Further investigations were carried out on samples with trisomy 21 for PRTs generated on chromosome 21. Of the six PRTs designed within Alu repeats, only one successfully called the correct copy number, whereas seven PRTs of fourteen designed within LTR and L1 elements produced clean products and successfully called copy number of chromosome 21 (An example can be seen in Supplementary Fig. S4). Overall, assays designed in LINEs, self-chain segments or other sequences differed minimally in success rate, with the main determinant being the potential number of amplicons generated through mispriming due to tolerance of nucleotide mismatches. Therefore, it is not recommended to design PRTs in SINES, although these could potentially be used with careful optimization, e.g. high annealing temperature, hot-start polymerase, short extension time and reduced cycle number.

### 3.3 Assay design across the human genome

Potential PRTs were designed across the human genome by partitioning chromosomes into 50 Mb intervals and running PRTPrimer on the University of Leicester high performance computing cluster. The parameters were set to design 2000 primer pairs per 2 kb

window (1 primer pair per nucleotide), a total of around 3 billion primer pairs. The total number of assays successfully designed was 65 604 294 (2.2% of primer pairs). Dividing the genome in 10 kb windows, 67% of windows with a GC content of between 30 and 50% contained a PRT, whereas 30% of windows with a GC content of >50% contained a PRT. Of the CNVs identified by the comprehensive study by Conrad *et al.* (2010), pre-designed PRT assays tagged 87% of CNVs >10 kb, 62% of CNVs between 5–10 kb and 16% of CNVs 0–5 kb. We conducted a detailed analysis of the features of the assays on chromosomes 13 and 19, which are gene-poor and gene-rich, respectively. Table 1 shows number of assays according to sequence or size difference between the target and reference amplicons, and whether the reference amplicon is >500 kb from the target (to aid in selecting references that are not in the same CNV).

**Table 1.** Counts of PRTPrimer designed assays for different criteria in two example chromosomes**:** chr13**,** which is gene**-**poo**r**, and chr19, which is gene-rich

PRT Criteria	Chromosome 19	Chromosome 13
Sequence difference	1 729 709	2 060 532
Paralogue <500 kb from target	1 051 948	391 476
Paralogue >500 kb from target	677 761	1 669 056
Paralogue >500 kb from target and size difference between amplicons >0 bp	359 020	863 971
Paralogue >500 kb from target and size difference between amplicons >2 bp	204 030	502 948

On chromosome 19 (55 994 806 bp excluding the centromere), there are 1 729 709 potential PRTs with a sequence difference between the amplicons, which fall into 11 335 contiguous clusters (average density one cluster per 4.9 kb). The mean gap between clusters is 4.7 kb, with a median of 0.8 kb. Apart from at the centromere, there are 32 gaps of over 100 kb (maximum gap 261 kb). Overall in any 50 kb window, there is an 84.5% chance of there being at least one cluster of PRT assays. Of the 1949 Ensembl genes (including RNA genes, pseudogenes etc) on chromosome 19, a total of 50.7% have at least one PRT assay within them, 81.2% have an assay within 10 kb, and 98.0% of genes have an assay within 50 kb. Conrad *et al.* found 71 CNV on chromosome 19, which were >10 kb, and of these 64 are tagged with a pre-designed PRT assay from this study (Conrad *et al.*, 2010).

On chromosome 13 (113 249 859 bp), there are 2 060 532 assays in 18 308 clusters (average density one cluster per 6.2 kb). The mean gap between clusters is 4.7 kb, with six gaps >100 kb (maximum 177 kb). In any 50 kb window, there is a 98.7% chance of there being at least one cluster of PRT assays. Of the 1177 Ensembl genes on chromosome 13, a total of 54.5% have at least one PRT assay within them, 92.5% have an assay within 10 kb, and 100% of them have an assay within 50 kb. Of the 41 CNV >10 kb on chromosome 13 identified by Conrad *et al*., 40 are tagged with a PRT assay from this study (Conrad *et al.*, 2010).

## 4 DISCUSSION

In this article, we describe a resource of off-the-shelf PRT assays as well as for custom design of assays. This resource, and PRTs in general, is conceived as an accessible method of validating and replicating CNV data generated by high-throughput approaches, such as array CGH, SNP genotyping arrays or genome-scale sequencing. PRT allows the accurate typing of copy number at individual genomic loci in hundreds of DNA samples at a low cost. The simplest form of PRT assays can be typed using PCR with unlabeled oligonucleotides and agarose gels, at a cost affordable by any laboratory. To increase throughput or reduce labor power, PRT assays can be multiplexed using capillary electrophoresis or by qPCR.

Previously the utility of the PRT had been limited by the difficulty of assay design. This has meant in practice that PRT has been used by a limited number of laboratories with experience of the design issues. PRTPrimer overcomes this limitation by allowing users to either use the pre-designed assays, or if no suitable ones are found, to run the software to generate custom assays. This facility should open up the use of PRT to a diverse range of laboratories that want type CNV in patient collections. The typical scenario we envisage is where array or sequencing data have identified a particular CNV as being possibly associated with a phenotype, and researchers want to type their own patient cohort for the same CNV without resorting to whole-genome analysis, whether because of affordability issues or lack of sufficient DNA. Large genomic laboratories may want to use PRT as an easy method of replicating high-throughput data by an alternative method with accurate quantification of copy number.

The software implementation of PRTPrimer involves a pipeline with genomic data passing through two well-established and publically available packages, Primer3 and isPCR, followed by data sorting, filtering and output. The approach relies on brute-force computation to design a high number of potential PCR products in a genomic region, align all of the primer pairs to the genome, using BLAT, to identify those that amplify exactly two products and then filter for those that have the desirable characteristics of a PRT assay. This approach contrasts with the current method of using genome annotation to guide assay design, allowing a far greater number of assays to be designed over a wider proportion of the genome.

Assays in the pre-designed database are not randomly distributed but fall into clusters of overlapping amplicons. Many of the amplicons within a cluster will vary by just a few base pairs at each end, and therefore do not represent truly different assays. However, other clusters will represent multiple assays that overlap. On a higher level the distribution of the clusters is also not random, with occasional gaps of >100 kb, although the mean gap is <5 kb. Of CNVs >10 kb, a high proportion is tagged by PRT assays in the database; smaller CNVs are less likely to contain a pre-designed PR. For quantifying gene copy number the ideal PRT assay will be between the first and last exons, a situation that exists for around half of genes, although 80–90% of genes have an assay within 10 kb. For smaller CNVs and the remaining 10–20% of genes, it may be possible to obtain assays using the software directly with altered parameters. Despite the high density of assays and clusters across the genome, there remain intractable parts of the genome where assay design is not possible, presumably because of the lack of suitable repeat elements. For targets in these limited regions, an alternative technology such as conventional qPCR will be needed. It should be noted that although the assays produced by PRTPrimer are bioinformatically predicted to produce two distinguishable amplification products, they require laboratory validation before use.

This resource is primarily focused on the human genome, but the underlying software can be easily adapted for other species with high genome coverage. We have successfully used PRTPrimer to design assays for two other mammalian species (mouse and rhesus macaque), but it could equally well be used for invertebrate or plant genomes. The authors are happy to advice researchers who would like to establish PRTPrimer for other genomes in their own laboratories.

*Funding*: This research was supported by Action Medical Research (grants SP4139 and SP4483), by the European Union’s Seventh Framework Programme (FP7/2007-2013) projects READNA (grant agreement HEALTH-F4-2008-201418) and GEN2PHEN (grant agreement number 200754), by the Medical Research Council New Investigator award (grant no. GO801123), by the Breast Cancer Campaign (2007NovPR45) and by Alzheimer’s Research UK Midlands Network Centre funding.

*Conflict of Interest*: none declared.

## Supplementary Material

Supplementary Data
