# Supplementary material for: Automated design of paralogue ratio test assays for the accurate and rapid typing of copy number variation
Source: Bioinformatics. 2013 Jun 6;29(16):1997–2003. doi: 10.1093/bioinformatics/btt330 (PMC3722521; doi:10.1093/bioinformatics/btt330)
Supplement: Supplementary Data [file supp_29_16_1997__index.html]

Automated design of paralogue ratio test assays for the accurate and rapid typing of copy number variation — Automated design of paralogue ratio test assays for the accurate and rapid typing of copy number variation — Supplementary Data 

# Automated design of paralogue ratio test assays for the accurate and rapid typing of copy number variation

## 

files

**Files in this Data Supplement:**

- Supplementary Data - docx file
