# Supplementary material for: Automated design of paralogue ratio test assays for the accurate and rapid typing of copy number variation
Source: Bioinformatics. 2013 Jun 6;29(16):1997–2003. doi: 10.1093/bioinformatics/btt330 (PMC3722521; doi:10.1093/bioinformatics/btt330)
Supplement: Supplementary Data [file supp_btt330_SupplementaryInfoVeal.docx]

**Supplementary Table 1 PRTPrimer parameters**

| **Command line and online** | | | | | |
| --- | --- | --- | --- | --- | --- |
| **Command** | **Short** | **Online** | **Parameters** | **Default** | **Description** |
| -jobname | -j | automatic | unique name |  | unique identifier of run |
| -seq |  | input boxes | chr start end |  | coordinates of target |
| -nosnpm |  | checkbox |  | 1 | no SNP masking |
| -noalum |  | checkbox |  | 1 | no Alu masking |
| -notrfm |  | checkbox |  | 1 | no TRF masking |
| -window | -w | input box | size(bp) | 2000 | size of windowing of target |
| -overlap | -o | input box | size(bp) | 300 | overlap between windows |
| -ppn |  | input box | numerical | 2 | primer pairs per nucleotide (average) |
| -prst |  | input box | size(bp) | 100 | minimum size of target product |
| -prend |  | input box | size(bp) | 300 | maximum size of target product |
| -pmin |  | input box | size(bp) | 18 | minimum primer length |
| -pmax |  | input box | size(bp) | 27 | maximum primer length |
| -popt |  | input box | size(bp) | 21 | optimum primer length |
| -minprod |  | input box | size(bp) | 0 | minimum size of reference product |
| -maxprod |  | input box | size(bp) | 500 | maximum size of reference product |
| -mindiff |  | input box | size(bp) | 3 | min size difference between target and reference |
| -maxdiff |  | input box | size(bp) | 500 | max size difference between target and reference |
| -dist |  | input box | size(bp) | 500000 | minimum distance between target and reference |
| -nhits |  | checkbox | numerical(max 5) |  | multiple targets option (-seq coordinate is first target) |
| -targetA |  | input box | chr start end |  |  |
| -targetB |  | input box | chr start end |  |  |
| -targetC |  | input box | chr start end |  |  |
| -targetD |  | input box | chr start end |  |  |
| **Command line only** | | | | | |
| **Command** | **Short** | **Online** | **Parameters** | **Default** | **Description** |
| -primer | -p |  | binary | 1 | option to not run primer3 |
| -ispcr | -i |  | binary | 1 | option to not run isPCR |
| -calculate | -c |  | binary | 1 | option to not process PRTs |
| -rerunispcr | -r |  | binary | 0 | option to rerun isPCR using input files |
| -sensitive | -s |  | binary | 0 | option to rerun using sensitive isPCR settings |
| -Allhits | -A |  | binary | 1 | outputs all data regarding number of hits in genome for all primer pairs |
| -local |  |  |  |  | uses local storage on a node of a cluster |
| -infile |  |  | text |  | fasta input |
| **Online only** | | | | | |
| **Command** | **Short** | **Online** | **Parameters** | **Default** | **Description** |
| Email |  | input box | text |  | sends results to address |

**Supplementary Figure 1**


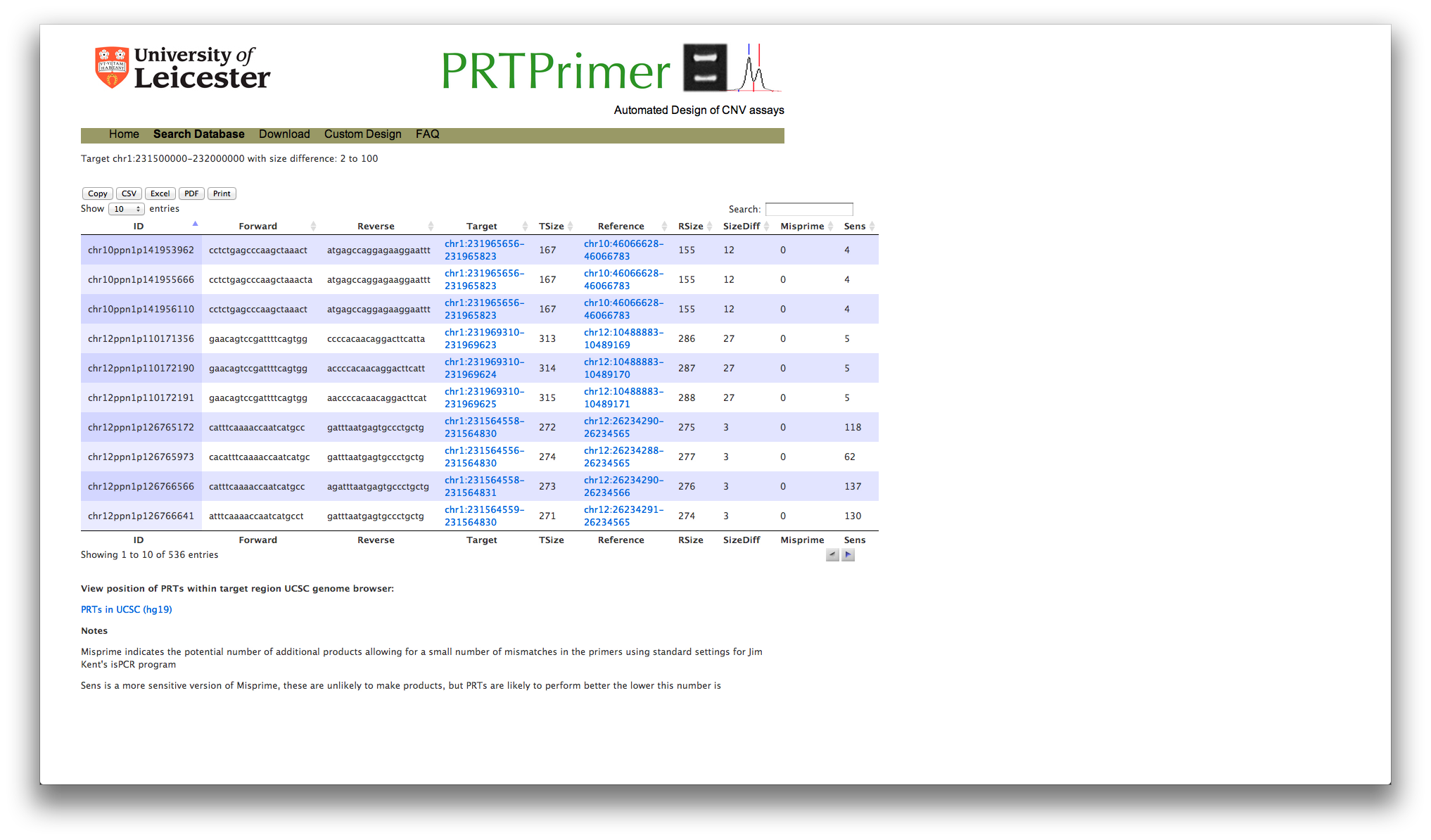


Example output from the *PRTPrimer* website for search of the pre-designed PRT database. The webpage includes information on the location and parameters used for the search, options for output to the local files and a multipage table of results, and a link to view the results on the UCSC genome browser. The table of results includes the ID, primers, the target and reference amplicons, size difference between amplicons, number of potential mis-priming sites under normal or sensitive settings.

**Supplementary Figure 2**


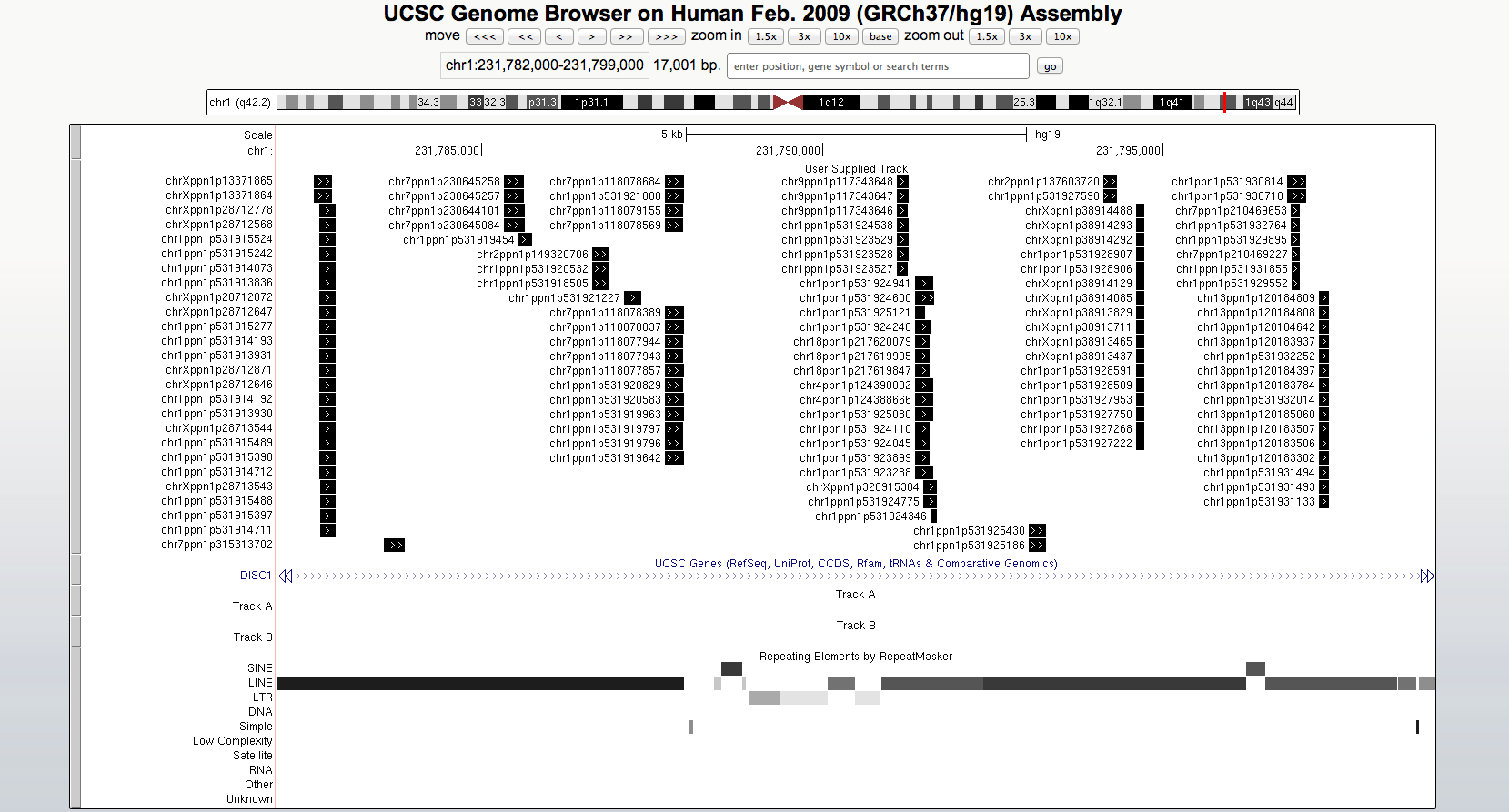


Example of the automated custom track generation for the UCSC genome browser. The PRT assays are plotted in a custom track in relation to other user selectable features. The ID and size of each PRT amplicon is displayed in relation to user selectable genome annotations. The view can be adjusted to collapse the PRT amplicons into a single line, or, as shown here, display each PRT amplicon individually.

**Supplementary Figure 3**

100bp

ladder 1 2 3 4 5 6 7 8 9 10 11 12(-ve)


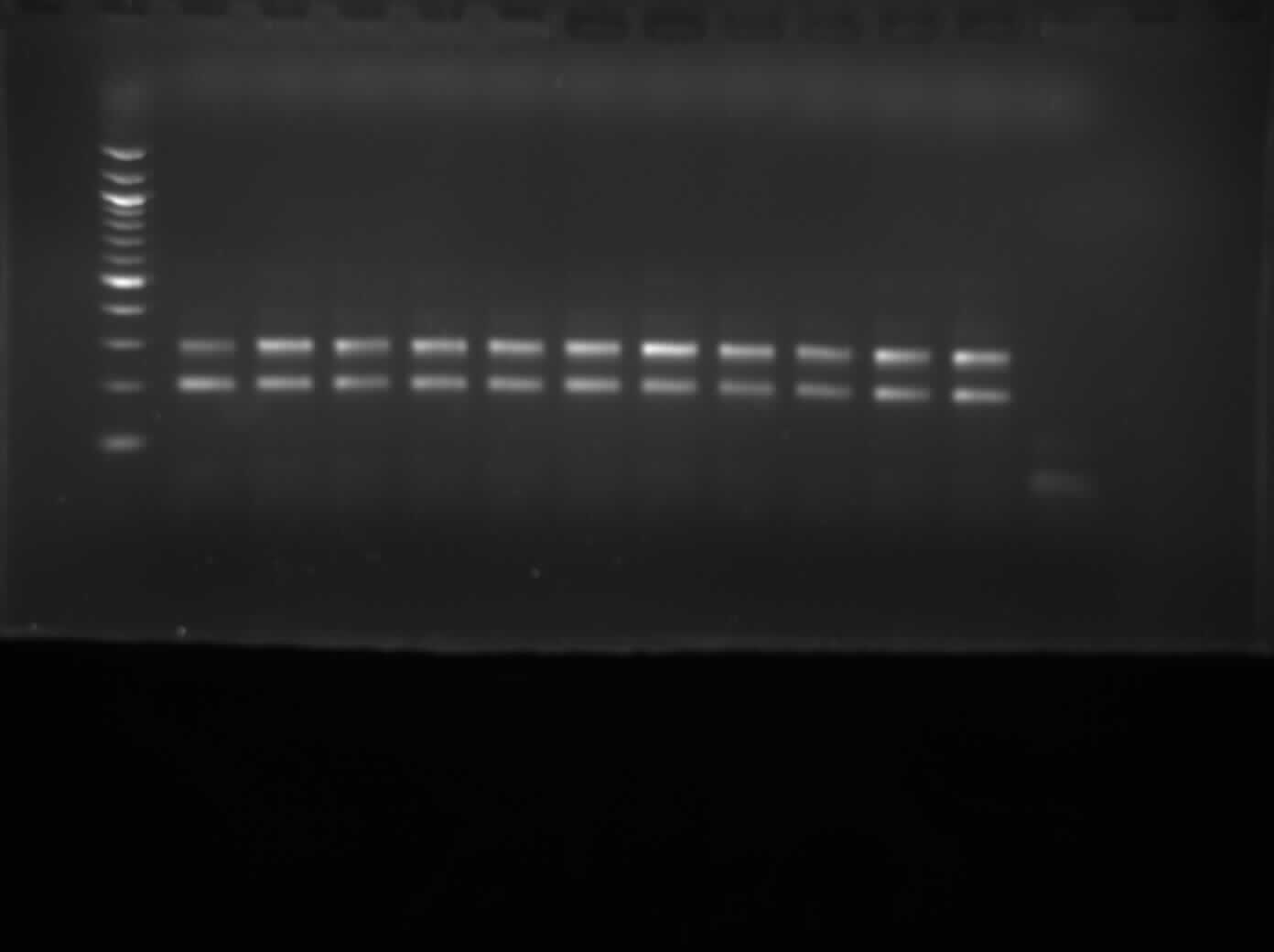


294 bp Chr 10

202 bp Chr 20

Top, an example gel of the two products generated by a PRT with the target on Chromosome 10 (294 bp) and the reference on chromosome 20 (202 bp). Bottom corresponding ratio between the two products, red indicates a deletion and blue indicates duplications.

**Supplementary Figure 4**

Ratio of the chromosome 21, target, and chromosome X, reference. Sample 7 is a trisomy 21 sample and has a ratio near the expected ratio of 1.5 whereas as all the non trisomy samples are closer to a ratio of 1.
